# Correction: Quantum chemical elucidation of the turn-on luminescence mechanism in two new Schiff bases as selective chemosensors of Zn^2+^: synthesis, theory and bioimaging applications

**DOI:** 10.1039/c9ra90081k

**Published:** 2019-11-04

**Authors:** Jessica C. Berrones-Reyes, Blanca M. Muñoz-Flores, Arelly M. Cantón-Diáz, Manuel A. Treto-Suárez, Dayan Páez-Hernández, Eduardo Schott, Ximena Zarate, Víctor M. Jiménez-Pérez

**Affiliations:** Universidad Autónoma de Nuevo León, Facultad de Ciencias Químicas, Ciudad Universitaria 66451 Nuevo León México victor.jimenezpr@uanl.edu.mx; Doctorado en Fisicoquímica Molecular, Center of Applied Nanosciences (CENS), Universidad Andres Bello Ave. República #275 Santiago de Chile Chile; Departamento de Química Inorgánica, UC Energy Research Center, Facultad de Química de Farmacia, Pontificia Universidad Católica de Chile Avenida Vicuña Mackenna, 4860 Macul Santiago Chile; Instituto de Ciencias Químicas Aplicadas, Theoretical and Computational Chemistry Center, Facultad de Ingeniería, Universidad Autónoma de Chile Av. Pedro de Valdivia 425 Santiago Chile ximena.zarate@uautonoma.cl; Millennium Nuclei on Catalytic Processes Towards Sustainable Chemistry (CSC) Chile

## Abstract

Correction for ‘Quantum chemical elucidation of the turn-on luminescence mechanism in two new Schiff bases as selective chemosensors of Zn^2+^: synthesis, theory and bioimaging applications’ by Jessica C. Berrones-Reyes *et al.*, *RSC Adv.*, 2019, **9**, 30778–30789.

The authors regret that an incorrect email address was given for affiliation d in the original manuscript. The correct email address is ximena.zarate@uautonoma.cl, and the corrected list of affiliations is as shown above.

The Royal Society of Chemistry apologises for these errors and any consequent inconvenience to authors and readers.

## Supplementary Material

